# Task modulation of brain responses in visual word recognition as studied using EEG/MEG and fMRI

**DOI:** 10.3389/fnhum.2013.00376

**Published:** 2013-07-23

**Authors:** Y. Chen, M. H. Davis, F. Pulvermüller, O. Hauk

**Affiliations:** ^1^Neuroscience and Aphasia Research Unit, University of ManchesterUK; ^2^MRC Cognition and Brain Sciences UnitCambridge, UK; ^3^Brain Language Laboratory, Department of Philosophy and Humanities, Freie Universität BerlinBerlin, Germany

**Keywords:** top–down control, lexical decision, semantic decision, reading, source estimation

## Abstract

Do task demands change the way we extract information from a stimulus, or only how we use this information for decision making? In order to answer this question for visual word recognition, we used EEG/MEG as well as fMRI to determine the latency ranges and spatial areas in which brain activation to words is modulated by task demands. We presented letter strings in three tasks (lexical decision, semantic decision, silent reading), and measured combined EEG/MEG as well as fMRI responses in two separate experiments. EEG/MEG sensor statistics revealed the earliest reliable task effects at around 150 ms, which were localized, using minimum norm estimates (MNE), to left inferior temporal, right anterior temporal and left precentral gyri. Later task effects (250 and 480 ms) occurred in left middle and inferior temporal gyri. Our fMRI data showed task effects in left inferior frontal, posterior superior temporal and precentral cortices. Although there was some correspondence between fMRI and EEG/MEG localizations, discrepancies predominated. We suggest that fMRI may be less sensitive to the early short-lived processes revealed in our EEG/MEG data. Our results indicate that task-specific processes start to penetrate word recognition already at 150 ms, suggesting that early word processing is flexible and intertwined with decision making.

## Introduction

Word recognition has long been thought to be largely automatic, as for example demonstrated in the classic Stroop effect (Stroop, 1992). Some authors have criticized this “curse of automaticity,” and pointed out that word processing should be considered as flexible, because behavioral performance in word processing tasks is highly task dependent (Balota and Yap, 2006). However, behavioral evidence alone cannot determine at which stage task demands modulate word processing. In one extreme, a visually presented word may automatically activate all types of information associated with it, but only the information relevant for the task is used for decision making. Alternatively, top–down control may already allow retrieval of specific types of word information that is necessary for the task. These two views are not mutually exclusive. A direct way of testing these views is to measure brain activation during on-line word processing: We can determine whether task demands modulate early or late stages of word processing, and whether the pattern of brain activation suggests different information processing at early and late stages. Here, we present data from a multi-modal imaging study, employing EEG/MEG and fMRI data from separate experiments, to investigate the effects of task demands on early word processing.

A number of studies have already demonstrated that information not essential for a task can still affect behavioral and brain responses, as demonstrated in the Stroop effect or in the effects of semantic variables in lexical decision (Chumbley and Balota, 1984; Kiefer and Spitzer, 2000; Neely and Kahan, 2001; Balota et al., 2004; Heil et al., 2004). However, the size of these effects has been shown to depend on task demands, demonstrating some degree of flexibility (James, 1975; Balota et al., 2004; Woollams, 2005; Evans et al., 2012). Although there is strong evidence that we cannot completely suppress the retrieval of task-irrelevant information, it is still not clear at what stage task demands affect word processing.

Behavioral data alone are inherently limited in their ability to distinguish different processing stages (Anderson, 1978). Only methods such as EEG and MEG enable us to record brain activation on-line, i.e., while word recognition is unfolding. Recent ERP studies have shown that task demands can affect the mechanisms of masked and unmasked priming. In a masked priming ERP study, the reduction of N400 amplitude to target words preceded by semantically-related prime words was greater if preceded by another semantic task, and smaller if preceded by a perceptual task (Kiefer and Martens, 2010). The authors suggest that different preceding tasks can either “sensitize” or “desensitize” the semantic system, causing the enhancement or inhibition of semantic processing of the masked prime, respectively. This interesting study still does not allow discrimination between the decision or retrieval views. The preceding task may affect the ease with which relevant information for the next task is retrieved, or the ease with which this information can be used for decision making. In our view, the most direct way to clarify this issue is to use single-word paradigms, and monitor the time course of early word recognition with high temporal resolution.

Unfortunately, the literature is still inconsistent with regard to even basic aspects of the time course of visual word recognition. In particular, the latency ranges for lexico-semantic information retrieval are still intensely debated, with estimates ranging from before 150 to about 350 ms (Pylkkanen and Marantz, 2003; Sereno and Rayner, 2003; Grainger and Holcomb, 2009; Pulvermuller et al., 2009; Kutas and Federmeier, 2011; Hauk et al., 2012). We will therefore present results for several latency ranges. However, we adopt the view that the earliest modulation of lexico-semantic information retrieval can be expected around the N170 component. A number of studies have reported effects of lexical and semantic variables in this latency range (Sereno and Rayner, 2003; Hauk and Pulvermuller, 2004a; Amsel, 2011; Van Doren et al., 2012; Amsel et al., 2013), and a recent study has provided consistent evidence from behavioral responses, ERPs and EEG/MEG source estimation for this assumption (Hauk et al., 2012). We assume that an absence of task effects before this latency (e.g., around 100 ms) would indicate that tasks are similar with respect to general attentional demands.

The “earliness” of task modulation in brain responses would already provide strong evidence as to whether they reflect differences in early information retrieval or late decision making. In addition, the pattern of brain activation at early and late latencies may reveal whether task demands modulate brain areas usually associated with executive functions, or with early lexico-semantic information retrieval. We therefore applied state-of-the-art distributed source estimation using the minimum norm method (Dale and Sereno, 1993; Hämäläinen and Ilmoniemi, 1994; Hauk, 2004) to our combined EEG and MEG data using individual head geometries.

fMRI cannot provide evidence about the timing of cognitive processes, but a comparison of fMRI localization and EEG/MEG source estimation results may provide better clues about the spatio-temporal dynamics of the neuronal generators. A number of brain regions, mainly in left perisylvian cortex, have been implicated in orthographic, lexical and semantic processing (Jobard et al., 2003; Binder et al., 2009). Interestingly, several studies have reported task effects mainly in frontal brain regions for visual word recognition (Chee et al., 2002; Fiebach et al., 2007). We therefore also asked whether fMRI can detect task differences in perisylvian regions commonly associated with early word processing, or whether it is mostly sensitive to later processes related to decision-making and response execution.

Although some studies on perceptual processes have shown good correspondence between metabolic neuroimaging and EEG/MEG activation (Heinze et al., 1994; Opitz et al., 1999; Sharon et al., 2007), combining EEG/MEG with fMRI for higher cognitive functions, in particular for language processing, has so far been less successful (Liljestrom et al., 2009; McDonald et al., 2010; Vartiainen et al., 2011). We therefore did not attempt a direct fusion of our EEG/MEG and fMRI data. Instead, we performed an independent analysis of EEG/MEG and fMRI data, in order to study their consistencies and discrepancies.

We employed three different psycholinguistic tasks that are commonly used in behavioral and neuroimaging research: (1) Lexical Decision (LexT), (2) Semantic Decision (SemT), and (3) Silent Reading (SilT). LexT responses can be made based on the general “wordlikeness,” i.e., do not require word identification, although a number of studies mentioned above have shown that semantic information is retrieved in LexT as well. SemT (e.g., deciding whether a word is a person's name) explicitly requires identification of an individual word and its meaning, and is therefore putting more emphasis on semantic processing. To avoid muscle and movement artifacts due to overt articulation in our EEG/MEG and fMRI data, we employed a silent reading task instead of reading aloud, which should still involve phonological processes. Silent reading has successfully been employed in several previous neuroimaging experiments (Joubert et al., 2004; Hauk et al., 2008b; Kronbichler et al., 2009). We focused mainly on the time course of brain activity as measured with EEG/MEG, since this is most informative with respect to top–down effects on early word recognition processes. We also compared our EEG/MEG results with fMRI data from a different participant group, in order to corroborate our EEG/MEG source estimation results, and to determine the differential sensitivities of EEG/MEG and fMRI to different aspects of visual word recognition.

## Methods

### Participants

Fifteen subjects (11 female) entered the EEG/MEG analysis after three subjects were removed due to excessive movement and eye blinking artifacts. Twenty subjects (10 female) entered the fMRI analysis after two subjects were removed due to measurement error. A reduced version of the Oldfield handedness inventory (Oldfield, 1971) showed no significant difference between EEG/MEG and fMRI participants in handedness (*M* of laterality Quotient = 86.9 vs. 86.8, *p* = 0.982), age (*M* = 25 vs. 25.7, *p* = 0.719), and there was no difference with respect to self-reported years of education (*M* = 16.6 vs. 17.3, *p* = 0.515). All participants were native English speakers, had normal or corrected-to-normal vision and reported no neurological disorder or dyslexia. They were paid 10 pounds per hour for their participation (a minimum of £20 for the whole experiment). The experiment was approved by the Cambridge Psychology Research Ethics Committee.

### Stimuli

Six hundred words (200 per task) were selected from the MRC psycholinguistic database based on the criteria that their word length ranged between 3 and 7 letters, their word form frequency and lemma frequency per million were greater than 0 and they were not listed as morphologically complex in the CELEX database (Baayen et al., 1993). Bigram frequency, trigram frequency, word length, word form frequency, lemma frequency, and neighborhood size (Coltheart's *N*) were obtained from CELEX database (Baayen et al., 1993). Number of semantic meanings and number of senses were obtained from the Wordsmyth database (http://www.wordsmyth.net/). Action-relatedness ratings of stimuli were obtained using the same method as Hauk et al. (2008a,b). Concreteness and imageability were obtained from the MRC psycholinguistic database. Stimuli were divided into three lists, matched on all the variables mentioned above using the Match software (van Casteren and Davis, 2007), as shown in Table 1. The three word lists were counterbalanced over the three tasks.

**Table 1:** **Mean values (and standard deviations) of 11 psycholinguistic variables for the three word lists and one pseudoword list**.

	**Word list 1**	**Word list 2**	**Word list 3**	**Pseudowords**
Word length	5.03(1.1)	5.05(1.04)	4.9(0.89)	4.86(0.79)
Bigram	32882.39(12136.29)	33855.98(13533.4)	34113.87(13828.06)	34392.19(14063)
Trigram	3568.2(2195.92)	3509.98(2244.32)	3394.78(2218.53)	3346.39(2155.28)
*N*	4.07(4.29)	4.17(4.21)	4.2(4.12)	4.21(4.05)
Word form frequency	41.92(76.59)	44.01(75.69)	42.62(73.91)	N/A
Lemma frequency	80.14(143.67)	88.06(204.32)	72.44(112.21)	N/A
Concreteness	511.19(107.62)	514.99(97.89)	517.81(105.73)	N/A
Imageability	524.69(86.01)	527.9(81.51)	529.33(84.18)	N/A
Action relatedness	3.31(0.95)	3.32(0.94)	2.82(1.2)	N/A
Number of meanings	1.15(0.4)	1.19(0.51)	1.18(0.45)	N/A
Number of senses	4.62(3.06)	4.96(3.14)	5.15(3)	N/A

Two hundred pseudowords were created for the lexical decision task. They were matched with the three lists of real words in word length, bigram frequency, trigram frequency and *N*. For the semantic decision task, twenty common person's names (e.g., Jack, Mandy) were selected as catch trials. Names were matched in word length (i.e., 3–7 letters) to the non-target words. Mix software (van Casteren and Davis, 2006) was used to randomize the pseudowords and real words in the lexical decision task, real words in silent reading, as well as real words and catch trial items in semantic decision. Words starting with the same letter did not follow each other in the experiment.

### Procedure

Each participant performed three psycholinguistic tasks: lexical decision task (LexT), silent reading task (SilT) and semantic decision task (SemT). The lexical decision task required participants to press buttons using the left hand middle finger for 200 pseudowords and the left hand index finger for 200 real words. The silent reading task required participants to silently read 200 words without making any overt articulatory response. The semantic decision task required participants to press a button using their left hand middle finger when they saw a target word corresponding to a person name. There were 20 target trials out of 220 total trials. Task order was counterbalanced across subjects in both fMRI and EEG/MEG.

For all three tasks, stimuli were presented for 100 ms, followed by a red fixation cross which had variable duration [*M* = 2400 ms, Range = (2150–2650)]. The average SOA was 2.5 s. Words were presented in a fixed width font (Courier New) in white on a black background. The longest word (7 letters) had a visual angle of 1.5° (fMRI) and 1.4° (EEG/MEG).

As the lexical decision was about twice as long as the other two tasks (due to the presence of pseudowords), it was split into two halves so that the whole experiment contained four blocks of comparable length. Breaks of 10 s were inserted after every minute of stimulus presentation. Each block lasted for 11 min except for the semantic decision task which was 12 min long due to the presence of 20 additional target trials. Before the first block of lexical decision task and the semantic decision task, a practice containing 10 items was given to the participants to ensure the task was well understood. As silent reading required no response inside the scanner, participants performed an unannounced post-scan word recognition test to ensure they had attended to the stimuli. In the recognition test, participants saw 40 words one at a time and were required to determine whether the words had been seen in the scanner using button presses. Half of the words had been presented previously and the other half were matched controls.

### EEG/MEG data acquisition and pre-processing

MEG data were acquired using a 306-channel Neuromag Vectorview system which contained 204 planar gradiometers and 102 magnetometers at MRC Cognition and Brain Sciences Unit, Cambridge, UK. EEG data were acquired simultaneously using a 70-electrode EEG cap (EasyCap), with the recording reference electrode attached to the nose, and the ground electrode to the left cheek. The electrooculogram (EOG) was recorded by placing electrodes above and below the left eye (vertical EOG) and at the outer canthi (horizontal EOG). To ensure accurate co-registration with MRI data, the positions of 5 Head Position Indicator (HPI) coils attached to the EEG cap, 3 anatomical landmark points (bilateral preauricular points and nasion), and 50–100 additional points covering the whole scalp were digitized with a 3Space Isotrak II System.

The signal-space separation (SSS) method implemented in the Maxfilter software (Version 2.0) of Neuromag was applied to the raw MEG data to remove noise generated from sources distant to the sensor array (Taulu and Kajola, 2005). In this process, movement compensation was applied and bad MEG channels were interpolated. Data acquired in all blocks except the first one were interpolated to the sensor array of the first block. Data were band-pass-filtered between 0.1 and 40 Hz using MNE software (Version 2.6) and downsampled to 4 ms time resolution. Data were divided into epochs of 600 ms, starting from 100 ms before stimuli onset. Epochs were rejected if maximum-minimum amplitudes in the −100 to 500 ms interval exceeded the following thresholds: 100 μV in the EEG, 100 μV in the EOG, 2500 fT in magnetometers, 1000 fT/cm for gradiometers. Raw data were inspected for each subject to check for consistently bad EEG channels, which were subsequently interpolated.

### fMRI data acquisition and pre-processing

Functional MRI scanning was performed using a Siemens 3T Tim Trio MR system with a head coil at the MRC Cognition and Brain Sciences Unit. Echo planar images (EPI) were acquired using a *TR* = 2 s, *TE* = 30 ms and a flip angle of 78°. Reconstructed images contained 32 slices covering the whole brain, with slice thickness 3 mm, interslice distance 0.75 mms, field-of-view 192 mm and in-plane resolution 64 × 64 voxels (3 × 3 mm). Functional scans were preceded by a high-resolution structural T1-weighted MRI images, acquired using a 3D MPRAGE sequence, field-of-view 256 × 240 × 160 mm, matrix dimensions 256 × 240 × 160, 1 mm isotropic resolution, *TR* = 2250 ms, *TI* = 900 ms, *TE* = 2.99 ms, flip angle 9°. Structural images were also acquired using the same sequence for participants in the EEG/MEG experiment.

Functional images were corrected for slice timing and realigned to the middle image. The EPI images were coregistered to the skull-stripped structural T1-images using a mutual information coregistration procedure (Pluim et al., 2003). The structural MRI was normalized to the 152-subject T1 template of the Montreal Neurological Institute (MNI). The resulting transformation parameters were applied to the coregistered EPI image. Images were resampled with a spatial resolution of 2 × 2 × 2 mm^3^, and spatially smoothed with a 10-mm full-width half-maximum Gaussian kernel. Low-frequency noise was removed by applying a high-pass filter (time constant 128 s). Imaging data was processed using SPM5 software (Wellcome Department of Cognitive Neurology, London, UK) and Automatic Analysis script (https://github.com/rhodricusack/automaticanalysis/wiki).

### Statistical analysis

#### EEG/MEG sensorspms and display

Statistical analysis of EEG/MEG amplitude in sensor space data was performed using “SensorSPMs” in SPM5. Two spatial dimensions of the sensor array and one dimension of time were combined into a 3D “volume,” and subjected to SPM analysis comparable to fMRI whole-brain analysis (http://imaging.mrc-cbu.cam.ac.uk/meg/SensorSpm). *F*-tests were computed at every latency and sensor. The significance threshold for the resulting *F*-distribution was determined using random field theory, taking into account the multiple comparisons problem across both space and time (Kiebel and Friston, 2004). SensorSPMs can only be computed for each sensor type separately (electrodes, magnetometers and gradiometers), due to different physical measurement units for the three sensor types. Furthermore, so far random field theory has only been applied to scalar fields (not vector fields), i.e., distributions that contain one value per location. For gradiometers, we could therefore combine the values of the two gradiometers at each location by computing the root-mean-square (RMS). However, this may produce spurious differences due to different noise-levels, as the RMS procedure produces only positive values which may turn differences in noise levels into differences in mean activations. We therefore only present SensorSPMs for magnetometer data and EEG. However, ROI statistics on source estimates (see below) are based on combined data from all sensor types.

In order to describe the time course of our data and to determine peaks and latency ranges of interest, we displayed the RMS of the signal-to-noise ratio (SNR) across all magnetometers, gradiometers and electrodes (dividing signal amplitude by the standard deviation of the baseline for each channel). The computation of SNRs prior to RMS transformation renders the values for all channels unit-less (original measurements are in T,T/m and μV, respectively), and allows the computation of a combined measure for display.

#### Source estimation of EEG/MEG data

Our source estimation procedure followed the standard procedure described for the MNE software (http://www.nmr.mgh.harvard.edu/martinos/userInfo/data/sofMNE.php). Minimum norm estimates (Hämäläinen and Ilmoniemi, 1994; Hauk, 2004) were computed on individually reconstructed cortical surfaces using boundary element models of the head geometry derived from structural MRI images. MEG sensor configurations and MRI images were co-registered based on the matching of about 50–100 digitized additional points on the scalp surface with the reconstructed scalp surface from the FreeSurfer software (Version 4.3; http://surfer.nmr.mgh.harvard.edu/). Structural MRI images were processed using the automated segmentation algorithms of FreeSurfer (Dale et al., 1999; Fischl et al., 1999). The noise covariance matrices for each data set were computed for baseline intervals of 200 ms duration before the onset of each stimulus presented during the whole experiment. For regularization, the default signal-to-noise ratio in the MNE software was used (*SNR* = 3).

The result of the FreeSurfer segmentation was processed further using the MNE software package (Version 2.6). The original triangulated cortical surface (consisting of several hundred thousand vertices) was downsampled to a grid using the traditional method for cortical surface decimation with an average distance between vertices of 5 mm, which resulted in ~10000 vertices. For MEG, a boundary element model (BEM) containing 5120 triangles was created from the inner skull surface using a watershed algorithm. For EEG, a three-layer BEM containing 5120 triangles were created from scalp, outer skull surface and inner skull surface respectively. Dipole sources were assumed to be perpendicular to the cortical surface. Source estimates were computed for each subject. The individual results were morphed to the average brain across all subjects, and a grand-average was computed. These grand-averages were then displayed on the inflated average cortical surface.

Six ROIs in regions commonly associated with word or language processing in the left hemisphere and their right hemisphere counterparts were defined on the basis of the All-words condition from EEG/MEG, which is orthogonal to the contrasts computed in the further analyses (Friston and Henson, 2006). It is important to note that spatial resolution of EEG/MEG data is inherently limited, and that source estimation may suffer from systematic mislocalization of the true sources (Fuchs et al., 1999; Molins et al., 2008; Hauk et al., 2011). Using standard coordinates from metabolic imaging studies, or from the fMRI part of our study, is therefore not recommended. Instead, we defined ROIs that fell within general areas of interest based on activation for all words averaged across all three tasks. This allows localization within the resolution limits of our sensor configuration, and ensures that ROIs are defined independently of the between-task comparisons and comprise parts of the source space to which our measurement configuration is sensitive.

**Figure 1:**
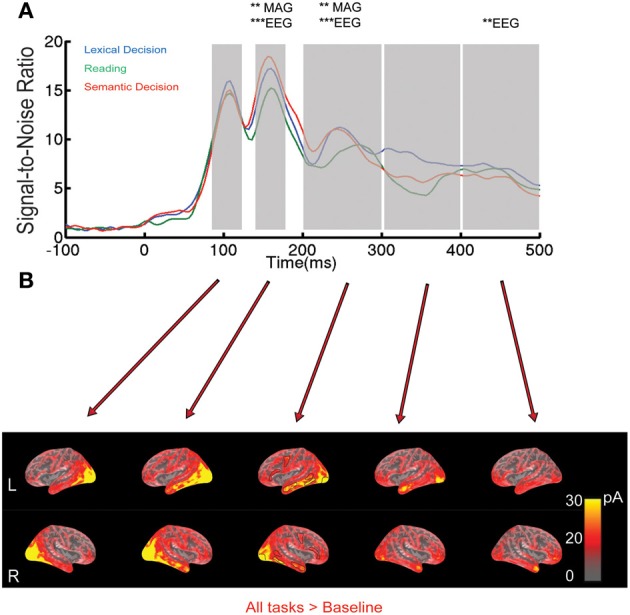
**EEG/MEG results for all words collapsed across the three tasks. (A)** Root-Mean-Square (RMS) of Signal-to-Noise Ratio (SNR, based on standard deviation of baseline interval) across all EEG/MEG sensors over time. The shaded areas indicate the five time windows selected based on peaks in the RMS curves: 92–124, 144–176, 200–300, 300–400, and 400–492 ms. Asterisks indicate whether a One-Way ANOVA with factor Task was significant at a family-wise corrected level (^**^p < 0.01, ^***^p < 0.001). MAG stands for magnetometers. **(B)** EEG/MEG source estimates on a group-averaged inflated brain surface for words averaged across tasks in the time windows specified above. ROIs selected for further analysis are outlined in black in the middle column.

General areas of interest were defined using anatomical labels provided in FreeSurfer software (e.g., “middle temporal cortex”). More precisely, for every general area of interest, we detected the most prominent activation peak within the time window 100–500 ms. We then extracted the ROI around this peak using the mne_analyze tool in the MNE software, approximately following the line of half-maximum-amplitude around the peak. In this way, six ROIs in each hemisphere were defined on the inflated surface as shown in Figure 1B. They were inferior lateral occipital gyrus, anterior middle temporal gyrus, precentral gyrus, inferior temporal gyrus, posterior middle temporal gyrus, and inferior frontal gyrus. For each ROI, average amplitudes within time windows and across vertices were computed. We performed Two-Way ANOVAs (factors Task and ROI) in the left hemisphere. We furthermore compared tasks in individual ROIs. Tasks were compared by means of pair-wise two-tailed t-tests in each ROI. We compared individual tasks to each other, i.e., LexT vs. SemT, LexT vs. SilT, and SemT vs. SilT, but also compared the mean of LexT and SemT (LexSemT) to SilT in order to increase statistical power to detect lexico-semantic effects.

#### fMRI analysis

fMRI analysis was conducted using the general linear model implemented in SPM5 software. Each stimulus event in each category (words, pseudowords, error trials for words and pseudowords in LexT, words and targets in semantic decision, words only in silent reading) was modeled as separate columns in the design matrix, and then convolved with the canonical haemodynamic response function (HRF) in SPM5. Covariates of no interest were added for the six movement parameters (translation and rotation) as well as the mean activation level within each session. Parameter estimates from the least mean square fit of the design matrix in each participant were entered into group analyses with inter-subject variation as a random effect (Holmes and Friston, 1998). For initial inspection of results, and selection of ROIs, the contrast of the response to all words in each task against the unmodeled resting period (i.e., implicit baseline) for each participant (i.e., All-words vs. Baseline) was used for analysis. Further contrasts between tasks were evaluated using one sample *t*-tests applied to subtractions of parameter estimates in each subject.

Depending on the research questions, several ROIs were used in different types of analyses, as will be described in the appropriate parts of the Results section. ROI analysis was performed using 4-mm-radius spheres centered at peaks as well as their corresponding right hemisphere peaks by flipping the sign of x coordinate in MNI space. Small volume correction (SVC) was performed in spheres of 15 mm radius around the corresponding peaks, or within labeled areas of the Automatic Anatomical Labeling (AAL) atlas (Tzourio-Mazoyer et al., 2002).

## Results

### Behavioral results

In LexT, the mean accuracy of detecting real words was 93% (*SD* = 4%) for EEG/MEG and 95% (*SD* = 4%) for fMRI. A Two-Way ANOVA with factors Method (fMRI vs. EEG/MEG) and Lexicality (words vs. pseudowords) revealed a significant effect of Lexicality (words vs. pseudowords in mean RT: 665 ms vs. 755 ms) [*F*_(1, 33)_ = 98.94, *p* < 0.001], but no significant effect of Method.

The mean *d*′ of post-scan word recognition tests in SilT was 0.94 (*SD* = 0.14) for EEG/MEG and 0.76 (*SD* = 0.15) for fMRI. Both were significantly different from 0 (EEG/MEG: *t* = 6.74, *df* = 14, *p* < 0.001; fMRI: *t* = 4.99, *df* = 19, *p* < 0.001). Because our participants were not instructed to memorize any of the stimuli prior to the experiment, we interpret these results as evidence that our participants attended to the stimuli during the experiment despite the absence of overt behavioral responses. The mean *d*′ for SemT in EEG/MEG was 4.36 (*SD* = 0.16) and in fMRI 4.30 (*SD* = 0.12). They were both significantly different from 0 (EEG/MEG: *t* = 27, *df* = 14, *p* < 0.001; fMRI: *t* = 36, *df* = 19, *p* < 0.001).

### EEG/MEG results

The mean numbers of trials rejected in the averaging process were 33.9 (*SD* = 29.8) (lexical decision), 27.7 (24.0) (silent reading) and 27.1 (27.5) (semantic decision). A One-Way repeated-measures ANOVA revealed no significant task difference in number of rejected trials [*F*_(2, 28)_ < 1, *p* > 0.5]. We analyzed our EEG/MEG data both in sensor and in source space. The results will be reported in separate sections below.

#### Sensor space

In order to illustrate the overall time course of brain activation, Figure 1A shows the root mean square (RMS) of signal-to-noise ratios (SNR) of all EEG/MEG sensors for words, separately for each task. In all three tasks, real words elicited clear peak responses around 100, 150, and 250 ms, with broader peaks around 350 and 450 ms. In order to test for reliable task differences in sensor space taking into account multiple comparisons across space and time, we employed a One-Way ANOVA with three levels (LexT, SemT, SilT) in a SensorSPM analysis, at a statistical threshold of *p* < 0.05 family-wise (FWE) voxel corrected with a minimum cluster size of 10 voxels. Based on SNR curves in Figure 1A, we tested within a time window from 92 to 500 ms. This analysis revealed the most reliable effect of task at around 150 ms (140 ms for magnetometers, *F* = 28.9, *p* < 0.01, FWE corrected; 152 ms for EEG, *F* = 46.58, *p* < 0.001, FWE corrected). *Post-hoc* pair-wise comparisons showed a significant difference between LexT and SilT in this latency range [magnetometers peaks at 144 ms, *F* = 26.71, *p* < 0.01, false discovery rate (FDR) corrected; EEG peaks at 176 ms, *F* = 85.49, *p* < 0.05, FWE corrected], as well as a significant difference between SemT and SilT (magnetometers peaks at 156 ms, *F* = 48.26, *p* < 0.01, FDR corrected; EEG peaks at 152 ms, *F* = 167.98, *p* < 0.001, FWE corrected) but no corrected significant difference between LexT and SemT.

Further significant effects were seen at 250 ms (magnetometers peak at 260 ms, *F* = 26.38, *p* < 0.01, FWE corrected; EEG peaks at 236 ms, *F* = 44.68, *p* < 0.001, FWE corrected). *Post-hoc* pair-wise comparisons revealed significant differences between LexT and SemT (magnetometers not significant; EEG peaks at 260 ms, *F* = 46.16, *p* < 0.05, FDR corrected), between LexT and SilT (magnetometer peaks at 256 ms, *F* = 43.08, *p* < 0.01, FDR corrected; EEG peaks at 232 ms, *F* = 113.61, *p* < 0.01, FWE corrected), and between SilT and SemT (magnetometers peaks at 244 ms, *F* = 59.81, *p* < 0.01, FDR corrected; EEG peaks at 240 ms, *F* = 39.71, *p* < 0.01, FDR corrected).

Later effects were detected in EEG only, at 496 ms (EEG, *F* = 27.59, *p* < 0.01, FWE corrected). *Post-hoc* pair-wise comparisons revealed that there were significant differences between LexT and SilT (EEG peaks at 496 ms, *F* = 52.48, *p* < 0.001, FDR corrected), between LexT and SemT (EEG peaks at 500 ms, *F* = 31.56, *p* < 0.05, FDR corrected) but not between SilT and SemT.

**Figure 2:**
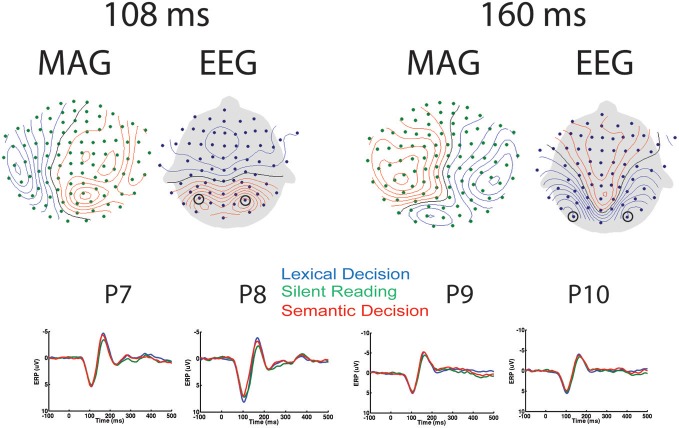
**EEG/MEG results in sensor space. Left:** Topography of EEG and Magnetometers (MAG) for All-word > Baseline contrast at 108 ms (P1), as well as time courses for peak electrodes (P7 and P8). **Right**: Topography of EEG and MAG for All-word > Baseline contrast at 160 ms (N1), as well as time courses for peak electrodes (P9 and P10). Contour line increments: 14fT for MAG, 0.85 μV for EEG.

Relatively few previous studies on word recognition have employed both EEG and MEG in the same experiment. It is therefore often difficult to compare MEG results with the usually more extensive ERP literature. Therefore, we present the topographies of EEG and magnetometer signals for All-word > baseline for two early latencies in Figure 2. In addition, time courses for peak electrodes are shown. This demonstrates typical posterior positive ERP components at 108 ms (“P1,” centre of 92–124 ms time window) and negative components at 160 ms (“N1,” center of 144–176 ms time window). The task effects revealed by the SensorSPM analysis (Figure 1) can also be seen in the ERP time courses for peak electrodes.

#### Source space

Our sensor space analysis provides strong evidence for task modulation of brain responses around 150 ms after word onset. In order to determine the likely neuronal generators that underlie these and later effects in sensor space, and compare them with fMRI results below, we computed minimum norm estimates from combined EEG and MEG data for the average across words for each task separately. Word-evoked activation for all words across tasks on inflated cortical surfaces is shown in Figure 1B. Time windows of 32 ms were used to capture the peaks (due to downsampling we had to choose a multiple of 4 ms). Strong bilateral occipital activation occurred in the 92–124 ms time window, followed by a strong widespread activation of the lateral and inferior portions of temporal lobe between 144 and 176 ms. In the 200–300 ms window, activation was more distributed, extending to regions such as left precentral and left inferior frontal cortex. Activation appeared mostly left-lateralized, especially in the anterior part of middle temporal region. In the 300–400 ms time window, activation started to diminish but was still present in occipital and anterior temporal lobe, and in the 400-492 ms time window in anterior temporal and frontal areas.

Task-differences were statistically analyzed using an ROI approach in the time windows defined above. Neuronal generators revealed by MNE were averaged within each specified time window.

As shown in Figure 1A, the signal-to-noise ratio curve peaks at 160 ms, and the most reliable effects in SensorSPMs occurred around this latency. We therefore focused our ROI analysis on the time window 144–176 ms. A Two-Way ANOVA [Task(3) × ROI(6)] in the left hemisphere revealed no effect of Task or a Task × ROI interaction (*p* > 0.6). On the basis of significant task effects in sensor space, we therefore carried out a more lenient analysis in source space analysis for individual ROIs, in order to provide an estimate for the most likely generators of our effects in sensor space. The reliability of these results will be discussed on the background of the existing neuroimaging literature.

**Figure 3:**
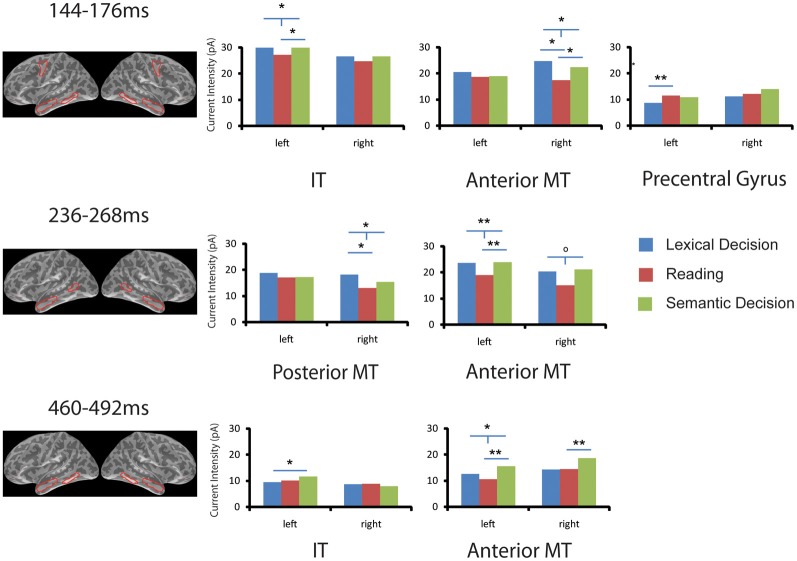
**ROI analysis in EEG/MEG source space.** Task effects on word activation are shown for six ROIs and in three time windows (144–176, 236–268, 460–492 ms) that showed significant family-wise corrected effects of Task in the sensor space analysis (SensorSPMs). For each time window, the anatomical locations of the ROIs with significant results are shown in red (^O^*p* < 0.1, ^*^p < 0.05, ^**^p < 0.01. “T”-bars indicate that combined lexical decision and semantic decision are different from silent reading). IT, inferior temporal gyrus; MT, middle temporal gyrus.

As shown in Figure 3, two-tailed paired sample *t*-tests showed that words in SilT elicited significantly stronger activation than those in LexT in left precentral gyrus [*t*_(14)_ = 3.141, *p* < 0.01] but not in the right hemisphere [*t*_(14)_ < 1]; SemT elicited stronger activation than SilT in left inferior temporal gyrus [*t*_(14)_ = 2.215, *p* < 0.05] but not in the right hemisphere [*t*_(14)_ < 1]. The average of LexT and SemT (LexSemT) showed greater activity than SilT in the left inferior temporal region [*t*_(14)_ = 2.208, *p* < 0.05]. SilT elicited weaker activation in right anterior temporal region than LexT [*t*_(14)_ = 2.693, *p* < 0.05] and SemT, although the latter effect was just marginally significant [*t*_(14)_ = 2.072, *p* = 0.057]. LexSemT showed greater activity than SilT in the right anterior temporal region [*t*_(14)_ = 2.550, *p* < 0.05] but not in the left anterior temporal cortex [*t*_(14)_ < 1].

The SensorSPM analysis suggested that further task effects occurred after the 150 ms time window. The Two-Way ANOVA (Task × ROI) in the left hemisphere in the 250 ms time window again revealed no task or task x ROI interaction (*p* > 0.2). For individual ROIs, SemT elicited stronger activation than SilT in left anterior temporal regions [*t*_(14)_ = 3.074, *p* < 0.01] but only marginally in the right anterior temporal region [*t*_(14)_ = 1.984, *p* = 0.067]. LexSemT showed greater activity than SilT in the left anterior temporal region [*t*_(14)_ = 3.053, *p* < 0.01], and marginally greater activity than SilT in the right anterior temporal region [*t*_(14)_ = 2.059, *p* = 0.059]. LexT elicited stronger activation than SilT in right posterior middle temporal region [*t*_(14)_ = 2.488, *p* < 0.05] but not in the left hemisphere [*t*_(14)_ < 1]. LexSemT showed greater activity than SilT in the right posterior middle temporal region [*t*_(14)_ = 2.516, *p* < 0.05].

In the time window 460 to 492 ms, the Two-Way ANOVA (Task × ROI) in the left hemisphere revealed a main effect of task (*p* < 0.05) but no Task x ROI interaction (*p* > 0.3). *Post-hoc* paired sample *t*-tests showed that the main effect of Task was due to a lower response in SilT than LexT (*p* < 0.05) and SemT (*p* < 0.01). In the individual ROI analysis, SemT again elicited stronger activation than SilT in left anterior temporal region [*t*_(14)_ = 3.077, *p* < 0.01] and in the right anterior temporal region [*t*_(14)_ = 3.187, *p* < 0.01]. LexSemT showed greater activity than SilT in the left anterior temporal region [*t*_(14)_ = 2.918, *p* < 0.05]. Furthermore, SemT elicited stronger activation than LexT in the left inferior temporal cortex [*t*_(14)_ = 2.291, *p* < 0.05] but not in the right hemisphere [*t*_(14)_ < 1].

### fMRI results

#### Whole-brain analysis

**Figure 4:**
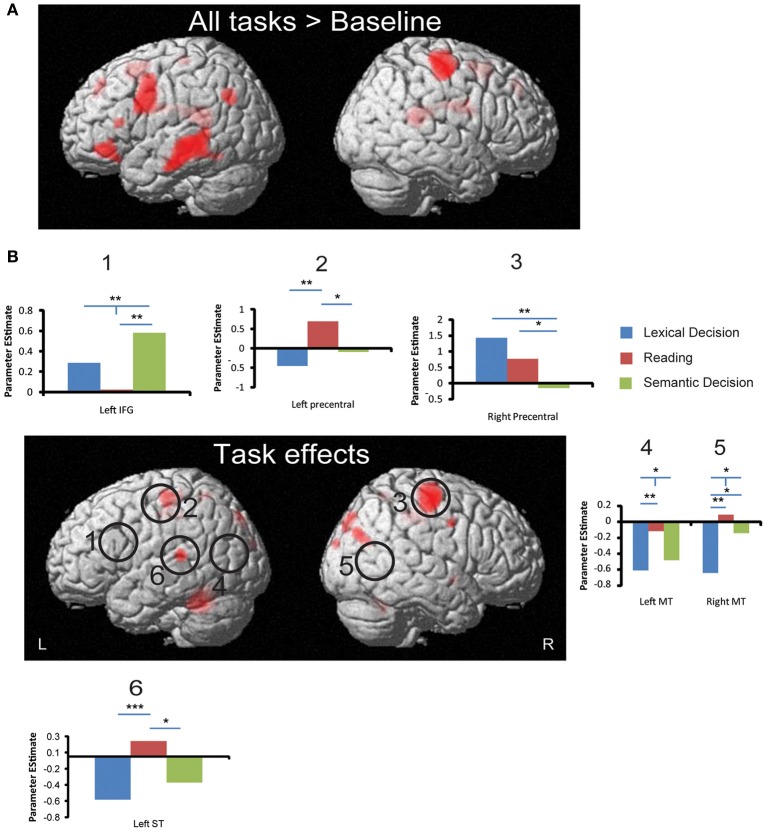
**fMRI activation to all words and task effects. (A)** Regions that produced significant activation to all words (averaged across three tasks) against baseline. **(B)** Regions that showed significant effects in a One-Way ANOVA with the factor Task. Circles indicate ROIs that showed significant task effects using small volume correction. Bar graphs show significant ROI analysis results in more detail (significance levels indicated as in Figure 3). All whole-brain results are displayed at a significance threshold of *p* < 0.001 uncorrected with minimum extent of 10 voxels (see Table 3 for details). ^*^p < 0.05, ^**^p < 0.01, ^***^p < 0.001.

As shown in Figure 4, real words against baseline across the three tasks elicited left lateralized activations in posterior cingulum, inferior temporal gyrus, angular gyrus, hippocampus, medial superior frontal gyrus and supplementary motor area in the left hemisphere, as well as right precentral gyrus, paracentral lobule and bilateral inferior frontal gyrus (see Table 2). It may surprise that we did not find activation in posterior inferior temporal and occipital areas for this contrast. However, this is likely due to the choice of our baseline task, which consisted of 10 s blocks of rest with instructions on the screen. Note that our task contrasts are orthogonal to the general word activation contrast, i.e., the absence of general word activation does not prevent us from detecting task effects.

**Table 2:** BOLD responses to words across tasks, at least *p* < 0.001 uncorr, minimum extent = 10, ^*^p < 0.05 FDR corrected, ^**^p < 0.05 FWE corrected.

**Region**	**Cluster**		***x***	***y***	***z***	***T***	***Z***
**WORDS IN ALL TASKS > BASELINE**							
L Post cingulum	3149	^**^	−16	−40	18	8.76	5.48
		^**^	20	−42	16	7.95	5.22
		^**^	−12	−2	24	7	4.87
L Inf temporal	1727	^**^	−42	−20	−20	7.49	5.05
		^**^	−48	−36	−4	7.31	4.98
		^**^	−46	−32	−14	7.2	4.94
R Precentral	673	^**^	36	−24	68	6.54	4.68
		^*^	42	−18	56	6.21	4.53
		^*^	44	−22	64	6.14	4.5
L Supp motor	219	^*^	−6	6	58	5.28	4.09
R Paracentral lobule	85	^*^	4	−28	62	4.83	3.85
L Inf frontal	283	^*^	−50	38	−8	4.65	3.75
		^*^	−40	30	−14	4.4	3.61
L Angular	75	^*^	−56	−64	36	4.49	3.66
L Hippocampus	48	^*^	−16	−12	−16	4.39	3.6
L Medial sup frontal	69	^*^	−10	40	42	4.12	3.44
L Inf frontal	48	^*^	−48	24	12	4.02	3.38
R Inf frontal	10	^*^	36	0	28	3.73	3.19

As shown in Figure 4, whole brain One-Way repeated measure ANOVAs with Task as the only factor revealed several regions that distinguished between the three tasks (*p* < 0.001, uncorrected, extent >10). At an FDR-corrected threshold, we found reliable task effects in cerebellum, superior temporal gyrus, precentral gyrus, middle cingulum and precueneus in the left hemisphere (see Table 3). In the right hemisphere task differences were seen in right precentral gyrus, middle cingulum, middle occipital gyrus, superior occipital gyrus, postcentral gyrus and pallidum. Among these regions, left superior temporal gyrus is important in language processing, especially for speech processing. The *post-hoc* ROI analysis centered at this peak (region 6 in Figure 4) showed that SilT elicited greater activation than LexT (*p* < 0.001) and SemT (*p* < 0.05). Further analysis in bilateral precentral gyrus is covered in pairwise comparisons later.

**Table 3:** Regions showing significant differences between tasks in One-Way repeated-measure ANOVA, at least *p* < 0.001 uncorr, minimum extent = 10, ^*^p < 0.05 FDR corrected, ^**^p < 0.05 FWE corrected.

**Region**	**Cluster**		***x***	***y***	***z***	***F***	***Z***
**ANOVA FOR THE EFFECT OF TASK**							
L Cerebellum	1019	^**^	−14	−52	−22	34.54	5.83
		^*^	10	−58	−34	9.52	3.32
R Precentral	677	^*^	40	−14	58	21.25	4.84
L Sup temporal	68	^*^	−62	−36	12	13.27	3.93
R Mid cingulum	173	^*^	14	−32	42	12.6	3.83
R Cuneus	166	^*^	16	−82	42	12.04	3.75
			30	−88	32	8.5	3.13
L Precentral	248	^*^	−36	−24	56	11.35	3.64
L Mid cingulum	178	^*^	−4	−22	46	11.09	3.6
		^*^	−10	−34	44	9.68	3.35
R Mid occipital	119	^*^	44	−76	30	11.07	3.59
		^*^	48	−70	24	10.39	3.48
L Precuneus	29	^*^	−10	−82	46	10.59	3.51
R Sup occipital	30	^*^	18	−94	22	10.37	3.47
R Pallidum	27	^*^	26	0	−8	9.66	3.35
R Postcentral	22	^*^	60	−2	38	9.59	3.34
L Precuneus	44	^*^	−6	−56	60	9.35	3.29

#### Small volume correction (SVC) and ROI analysis

Our whole-brain ANOVA did not reveal reliable task effects in areas that have previously been implicated in word processing including inferior frontal, middle temporal, inferior temporal, and precentral areas (Price, 2000; Jobard et al., 2003; Binder et al., 2009). Because some of these areas showed effects in our EEG/MEG source space analysis, we performed a more lenient analysis of our fMRI results based on small volume corrections (SVC) and ROIs. For SVC analyses, only results that survived *p* < 0.05 cluster correction are reported below.

Three peaks in left IFG and one peak in right IFG were revealed by whole brain All-word > Baseline activation contrast (*p* < 0.001 uncorrected, extent >10). SVC surrounding these peaks and their corresponding coordinates in the other hemisphere (by flipping the x coordinate) was performed for each of the pair-wise task comparisons. This revealed a significant cluster peaking in the left IFG (−36 14 26; *Z* = 3.25) for SemT > SilT (*p* < 0.05 cluster corrected). This result was confirmed by an SVC analysis using left pars triangularis AAL label. An SVC analysis using left pars opercularis AAL labels also revealed a cluster peak for SemT > SilT (−34 16 26; *Z* = 3.18) (*p* < 0.05 cluster corrected).

For middle temporal cortex, only SVC using bilateral AAL labels revealed for SilT > LexT significant clusters peaking in left posterior middle temporal cortex (−42 −68 22; *Z* = 3.49) (*p* < 0.05 cluster corrected), and right middle temporal cortex (52 −64 8; *Z* = 3.86) (*p* < 0.01 cluster corrected). No task differences were found in left inferior temporal cortex.

The All-word > Baseline contrast revealed a cluster in the right precentral region. ROI analysis centered on its peak showed that LexT elicited stronger activation than SemT (*p* < 0.001), and SilT elicited greater activation than SemT (*p* < 0.05) (region 3, Figure 4B). SVC analysis showed that SilT elicited greater activation than LexT in the left precentral gyrus (−36 −28 60; *Z* = 3.83) (*p* < 0.001 cluster corrected). In the right precentral gyrus, there was also a significant effect of LexT > SemT (38 −16 64; *Z* = 4.72) (*p* < 0.001 cluster corrected), and a significant effect of SilT > SemT (40 −28 58) (*p* < 0.05 cluster corrected). Finally, SVC analysis using bilateral AAL precentral gyrus labels revealed that SilT > LexT showed significant voxels in left precentral gyrus (−36 −28 60; *Z* = 3.83) (*p* < 0.01 cluster corrected). Right precentral gyrus showed elicited responses in LexT>SemT (40 −14 56; *Z* = 4.75) (*p* < 0.001 cluster corrected) and SilT>SemT (40 −26 56; *Z* = 3.51) (*p* < 0.05 cluster corrected) contrasts.

## Discussion

In order to investigate the flexibility of visual word recognition, we asked in which latency ranges and cortical areas brain responses are modulated by task demands. We studied word-evoked brain activity in three different psycholinguistic tasks using EEG/MEG and fMRI. Words were presented in lexical decision (LexT), silent reading (SilT) and semantic decision (SemT) tasks. Sensor space analysis of our EEG/MEG data revealed reliable task differences already around 150 ms after word onset. At this latency, task effects on brain activation in source space occurred in left inferior temporal, left precentral and right anterior temporal areas. These results suggest that task demands penetrate word processing already at an early stage. Left anterior temporal areas also showed task effects around 250 ms, and bilaterally around 480 ms. Within these time ranges, LexT and SemT produced more activation than SilT, except in precentral cortex. Around 480 ms SemT elicited greater activity than either LexT or SilT in bilateral anterior temporal lobes. The fMRI analysis revealed brain areas affected by task demands in left posterior superior temporal gyrus, bilateral precentral gyrus and left inferior frontal gyrus. This only partly matched our EEG/MEG results, and we suggest that fMRI may be more sensitive to later stages of word processing.

### EEG/MEG results

The main results of this study were early task effects in latency ranges around 150 and 250 ms after stimulus onset. This was revealed by statistical sensor space analysis that controlled for multiple comparisons across sensors and time samples (SensorSPM). Our tasks differed with respect to whether experimental sessions contained only words (SilT and SemT) or also pseudowords (LexT), and whether overt responses were required for every item (LexT), for a subset of items (SemT), or not at all (SilT). Similar differences also existed in previous behavioral studies, for example reading aloud and lexical decision will necessarily differ with respect to response selection. These studies usually employ block designs with respect to the factor Task, since randomizing tasks across trial would introduce potential confounds with regard to task switching. However, it is still possible that general differences in attention or response selection may explain our early task effects at around 150 ms. Considering that we did not find reliable task effects around 100 ms, and in particular that overall activity did not differ, we conclude that general attentional demands were similar across tasks.

We further asked whether there were specific differences between tasks in source space, i.e. whether the spatial pattern of our early task effects is consistent with the view that tasks already modulate early information retrieval. Surprisingly few neuroimaging studies have investigated task effects on general visual word recognition processes yet, and to our knowledge no results from comparable EEG/MEG source estimation studies are available. In the following, we will begin with a discussion of our novel EEG/MEG results, and then compare them with our fMRI data and previous fMRI literature.

The earliest task effects occurred around 150 ms, with more activation for SilT compared to LexT in precentral gyrus, as well as more activation to LexT and SemT compared to SilT in left inferior temporal cortex and right anterior middle temporal gyrus. Early activation of left precentral areas is consistent with early retrieval of phonological information, as reported in several previous studies. Pammer et al. (2004) found left precentral gyrus activation in the time window between 0 and 200 ms for words but not anagrams. Wheat et al. (2010) reported pseudohomophone priming effects in left precentral gyrus around 100 ms. This supports the view that silent reading (as an artifact-minimizing version of reading aloud) puts more emphasis on phonological information retrieval than LexT and SemT.

The localization of the task effect in left inferior temporal cortex around 150 ms is consistent with the “visual word form area” (VWFA), which is assumed to link higher-level orthography with lexical information (Cohen et al., 2000, 2002, 2004; Kronbichler et al., 2007; Dehaene and Cohen, 2011). Previous ERP studies have reported effects of lexical variables around 160 ms (Hauk et al., 2006a), and orthographic and lexical variables have been shown to interact around 160 ms (Hauk et al., 2006b). We therefore propose that task effects around 150 ms in the present study occurred at the earliest stage when orthographic and lexico-semantic information retrieval interact.

This conclusion is further supported by an early task effect in right anterior mid-temporal lobe (ATL). ATL is of particular interest with respect to semantics, since it has been labeled the “semantic hub,” i.e., it is thought to link word forms with distributed polymodal semantic representations (Patterson et al., 2007; Pulvermuller et al., 2010). The ATL has been shown to activate more under conditions where demands on the semantic system are increased (Woollams et al., 2011). It is possible that we missed a left-hemispheric effect around 150 ms due to a lack of sensitivity: ATL is located at the lower level of the EEG/MEG electrode and sensor array, and may therefore produce lower signals than for example more posterior and superior temporal areas. Differences in orientation or distribution of sources may have favored the right hemisphere over the left. Around 250 ms, a similar pattern of results can be observed in bilateral ATL. Our results at 150 ms may have captured the onset of this effect.

Greater activation for LexT and SemT than SilT in bilateral ATL around 250 ms, which was more reliable in the left hemisphere, indicates larger emphasis on semantic information retrieval in lexical and semantic tasks compared to silent reading. This was accompanied by a similar pattern in right posterior middle temporal gyrus, and is consistent with other studies that have reported effects of semantic variables in this latency range (Hauk and Pulvermuller, 2004b; Amsel, 2011). ATL also showed task effects around 480 ms in the left hemisphere, but with a different pattern: SemT activated more than LexT and SilT. Effects in this latency range (N400) are traditionally linked to semantic or conceptual processing, and ATL is one of the possible contributors to this components (Lau et al., 2008). We offer two possible explanations for the temporal sequence of our ATL effects. First, the later effect may be due to reevaluation of semantic information retrieved at earlier stages, which is necessary for accurate semantic decisions, but not for lexical decisions or silent reading. Second, it may reflect the time course of semantic information retrieval from coarse features early on to more fine-grained features at later latencies. A recent ERP study has demonstrated that different semantic features can modulate the brain response at different latencies (Amsel, 2011). A more detailed analysis of the time course of semantic processing using combined EEG/MEG source estimation should be provided by future studies.

### Comparison of EEG/MEG and fMRI results

Areas reliably modulated by task demands in fMRI were bilateral precentral gyrus, left superior temporal gyrus, bilateral posterior middle temporal lobe, and left inferior frontal gyrus. Among those areas, only left precentral gyrus showed task effects around 150 ms in our EEG/MEG analysis, with more activation for SilT compared to LexT. This corresponds well to our fMRI results, where left precentral gyrus was also more activated for SilT than for LexT. As described above, this is also consistent with the idea of early phonological activation in word reading (Pammer et al., 2004; Wheat et al., 2010), and may reflect grapheme-to-phoneme conversion in single-word reading (Jobard et al., 2003).

An area that showed a task effect in fMRI (more activation for SemT compared to SilT) but did not have a counterpart in our EEG/MEG analysis was the left inferior frontal gyrus (LIFG). This is consistent with a number of fMRI studies that have reported an involvement of LIFG in language processing, and semantics in particular (Bookheimer, 2002; Devlin et al., 2003; Jobard et al., 2003; Binder et al., 2009; Whitney et al., 2011). LIFG has also been shown to be sensitive to selection demands (Thompson-Schill, 2003), which would explain why it activates most in SemT where words and their meanings have to be uniquely identified. However, our EEG/MEG analysis did not reveal any task effects in this area in any of the analyzed latency ranges. Furthermore, our results presented in Figure 1B do not suggest strong activation in inferior frontal areas even to words in general within the first 500 ms. Note that we used combined EEG and MEG measurements in our source estimation procedure, which should have increased our chances to pick up signals in frontal areas compared to MEG alone (Molins et al., 2008; Goldenholz et al., 2009).

Further effects, for which a clear correspondence between EEG/MEG and fMRI could not be established, occurred in the posterior middle temporal lobe (pMTL). EEG/MEG detected task effects in pMTL only in the right hemisphere around 250 ms, where LexT produced more activation than SilT. In fMRI, the effects were reversed: SilT activates more than LexT. It is possible that the ROIs in the EEG/MEG and fMRI analyses reflect activation from different anatomical regions. For example, pMTL has been implicated in processing intelligible speech (Scott et al., 2000; Davis and Johnsrude, 2003). Higher activation in SilT might reflect stronger phonological processing or grapheme-to-phoneme conversion when participants were reading the word than performing LexT. This phonological account would also be consistent with the results that SilT activated left superior temporal gyrus more than both LexT and SemT. Superior temporal gyrus, together with posterior middle temporal gyrus and precentral gyrus, have been proposed to form a loop that is important for speech production (Scott et al., 2000). This is consistent with our assumption that the silent reading task encourages internal articulation and the retrieval of phonological information. Our fMRI data do not allow conclusions about the stage at which phonological information becomes available during word recognition. The fact that left pMTL effects were present in fMRI but not EEG/MEG suggests that they may reflect internal vocalization at a post-recognition stage.

Task effects in left inferior temporal cortex were found only in EEG/MEG, but not fMRI. They occurred in one early (150 ms) and one late (480 ms) latency range. As discussed earlier, left inferior temporal activation is often interpreted as reflecting early analysis of orthographic structures (Vinckier et al., 2007; Dehaene and Cohen, 2011). However, it has also been found to be modulated by variables other than orthography (Price and Devlin, 2011). In particular, several studies have reported imageability effects in left fusiform areas (Wise et al., 2000; Fiebach and Friederici, 2004; Sabsevitz et al., 2005; Hauk et al., 2008a), although some studies have failed to confirm these findings (Jessen et al., 2000; Binder et al., 2005). It is possible that these left inferior temporal brain areas are affected by multiple variables at multiple stages of processing. Indeed, our EEG/MEG data suggest different patterns of results at early and late latencies. It is therefore possible that different tasks activate inferior temporal areas at different stages and for different reasons, which in an fMRI analysis that integrates information over time may produce similar levels of activation.

The ATL is of particular interest with respect to semantics (Patterson et al., 2007; Pulvermuller et al., 2010). Unfortunately, fMRI is relatively insensitive to ATL activation due to effects of magnetic susceptibility (Devlin et al., 2000; Visser et al., 2009). Indeed, we did not find task effects in ATL in our fMRI data, but in three time windows of the EEG/MEG analysis: around 150 ms, 250 ms and 480 ms. It was first modulated by task demands in the right hemisphere (150 ms), followed by an effect in the left hemisphere (250 ms), and finally bilaterally (480 ms). Our results suggest that combined EEG/MEG source estimation is sensitive to ATL activation at different stages of processing, and may therefore be a promising tool for future studies in this area.

## Conclusions

In conclusion, our signal space EEG/MEG results indicate that task demands can already penetrate early stages of visual word processing. Furthermore, the patterns of brain activation obtained from distributed source estimation, which demonstrate the specificity of task effects to particular cortical regions, provide evidence that these task effects originate at early stages of lexico-semantic information retrieval. Our results do not contradict findings that task-irrelevant information can affect task performance (as in the Stroop effects); instead, in combination with previous results, we argue that visual word recognition is best described as flexible rather than automatic, and that information accumulation and decision making may be more intertwined than previously thought (Balota and Yap, 2006; Norris, 2006). An integration of these findings with our fMRI results is not straightforward, which we suggest reflects the relative insensitivity of fMRI to early short-lived processes that occur within the first 250 ms of word onset. Our results highlight the importance of EEG/MEG methodology in combination with source estimation for the investigation of the spatio-temporal dynamics of word recognition.

### Conflict of interest statement

The authors declare that the research was conducted in the absence of any commercial or financial relationships that could be construed as a potential conflict of interest.
